# Integrative DNA methylation and gene expression analysis to assess the universality of the CpG island methylator phenotype

**DOI:** 10.1186/s40246-015-0048-9

**Published:** 2015-10-13

**Authors:** Matahi Moarii, Fabien Reyal, Jean-Philippe Vert

**Affiliations:** CBIO-Centre for Computational Biology, Mines Paristech, PSL-Research University, 35 Rue Saint-Honore, Fontainebleau, F-77300 France; Department of Bioinformatics, Biostatistics and System Biology, Institut Curie, 11-13 Rue Pierre et Marie Curie, Paris, F-75248 France; U900, INSERM, 11-13 Rue Pierre et Marie Curie, Paris, F-75248 France; UMR932, Immunity and Cancer Team, Institut Curie, 26 Rue d’Ulm, Paris, 75006 France; Department of Translational Research, Residual Tumor and Response to Treatment Team, Institut Curie, 26 Rue d’Ulm, Paris, 75006 France; Department of Surgery, Institut Curie, 26 Rue d’Ulm, Paris, 75006 France

## Abstract

**Background:**

The CpG island methylator phenotype (CIMP) was first characterized in colorectal cancer but since has been extensively studied in several other tumor types such as breast, bladder, lung, and gastric. CIMP is of clinical importance as it has been reported to be associated with prognosis or response to treatment. However, the identification of a universal molecular basis to define CIMP across tumors has remained elusive.

**Results:**

We perform a genome-wide methylation analysis of over 2000 tumor samples from 5 cancer sites to assess the existence of a CIMP with common molecular basis across cancers. We then show that the CIMP phenotype is associated with specific gene expression variations. However, we do not find a common genetic signature in all tissues associated with CIMP.

**Conclusion:**

Our results suggest the existence of a universal epigenetic and transcriptomic signature that defines the CIMP across several tumor types but does not indicate the existence of a common genetic signature of CIMP.

**Electronic supplementary material:**

The online version of this article (doi:10.1186/s40246-015-0048-9) contains supplementary material, which is available to authorized users.

## Background

Epigenetic modifications have been recognized as important players in cancer etiology and development and constitute promising therapeutic targets for diagnosis or treatment due to their possible reversibility [1–3]. In particular, aberrant methylation of CpG islands (CGIs) located in promoter regions of tumor suppressor and DNA repair genes, leading to their silencing, is now considered a hallmark of cancer playing an important role in neoplasia [1–6].

The CpG island methylator phenotype (CIMP) was first defined and observed by [7] in a subset of colorectal cancers as the joint methylation of several promoter regions, leading to the inactivation of the corresponding genes. The stratification of patients based on CIMP was shown to be clinically relevant, as CIMP-positive patients had better prognosis than CIMP-negative ones, and could lead to personalized treatments. Since the identification of CIMP in colorectal cancers, many studies have tried to replicate the analysis to find CIMP in different types of cancers including but not limited to colon [8–12], breast [13, 14], lung [15], stomach [16], and glioblastoma [17–19]. While most of these works concluded in the existence of a CIMP in different cancers, other studies did not yield the same conclusions [20, 21], and the genes whose promoter CGI methylation are considered to define the CIMP differ between studies. This raises the question of whether the CIMP is tissue specific or is a universal phenomenon with common biological causes affecting common genes across cancers. A recent review of CIMP-related studies across different cancers pointed out the diversity of methods and measurement technologies used to define CIMP, which hinders the establishment of a molecular basis for CIMP in spite of growing evidence linking mutations in specific genes and CIMP in several cancers [22].

In the present study, we investigate the existence and universality of CIMP by performing a systematic genome-wide methylation analysis on several large datasets of different cancer types simultaneously. We propose a simple methodology to assess the existence of a CIMP phenotype in each cancer and to identify a set of genes whose promoter methylation is a marker for the CIMP. This allows us to compare the different cancer types in search for a cross-cancer CIMP signature and to analyze the link between CIMP and gene expression in different cancers. Finally, we assess the clinical relevance of CIMP on the overall survival.

## Results

### A cross-cancer CIMP signature

We first assess with a common methodology whether a CIMP can be detected on different cancers and whether CIMP in different cancers share a common signature in terms of which gene promoters are hypermethylated in CIMP-positive patients. For that purpose, we collected high-density methylation datasets from the cancer genome atlas (TCGA) data portal providing more than 485,000 CpG methylation levels for more than 2000 samples from five tissues of origin: bladder, breast, colon, lung, and stomach (Table 1). For each sample, we aggregate the methylation levels of CpG probes by CGI, including the CGI itself and its shores and shelves, resulting in a single methylation level for each of 21,176 CGIs in each sample.

**Table 1:** Patients’ dataset. Number of samples available for the different cancer types (first column) for methylation (Meth) and gene expression (GE). The “Meth/GE” column summarizes the number of samples with both methylation and gene expression, while the “Meth/Mutations” column shows the number of samples with both methylation and DNA mutation data

Tissue	Meth	GE	Meth/GE	Meth/Mutations
Bladder	373	56	*43*	*28*
Breast	626	778	*478*	*468*
Colon	291	193	*34*	*219*
Lung	452	125	*82*	*411*
Stomach	338	373	*309*	*199*
Overall	*2090*	*1525*	*941*	*1325*

A CIMP corresponds to the joint hypermethylation of a subset of CGIs in a subset of samples [7]. To characterize from whole-genome methylation data whether a CIMP exists for a cancer and which CGIs characterize it, we follow a standard methodology: (i) select the 5 % most variant CGIs in the set of samples, which we call the *CIMP signature* and (ii) check by unsupervised classification whether the samples cluster into two main clusters (CIMP-positive and -negative clusters) when we restrict them to the methylation values they take on the CGIs in the CIMP signature.

We apply this methodology to each of the five families of tumors, cutting the tree obtained by hierarchical clustering to two clusters in order to enforce a classification of all samples into two subgroups based on the methylation of CGIs in the CIMP signature. Interestingly, in all five cases, one of the two clusters is clearly characterized by an overall hypermethylation of most CGIs in the signature compared to the second cluster, allowing us to characterize it as the CIMP-positive cluster, the second one being the CIMP-negative cluster (Additional file 1). The proportion of CIMP-positive samples according to this definition varies from about 20 % for breast and colon cancers to 30 % for bladder and about 60 and 70 % for stomach and lung cancers (Table 2). Proportion of the CIMP-positive group in each tissue is similar to previously reported studies [22]. Varying the size of the CIMP signature from 1 to 10 % of all CGIs had a small impact on the clustering stability (Additional file 2).

**Table 2:** CIMP proportion. For each cancer type, this table shows the number of samples clustered in the CIMP-negative and CIMP-positive clusters and the percentage of CIMP-positive samples

Tissue	Negative	Positive	Ratio (%)
Bladder	262	111	30
Breast	509	117	19
Colon	232	59	20
Lung	136	316	70
Stomach	144	194	57
Overall	*1283*	*797*	*38*

**Fig. 1:**
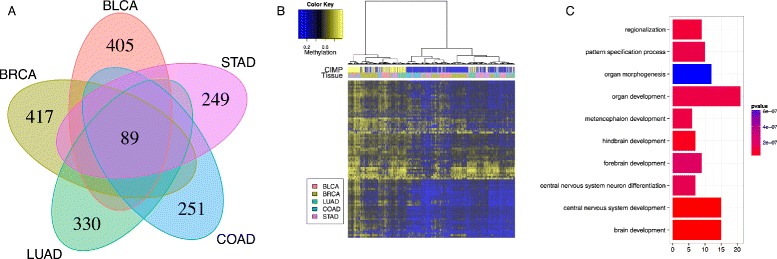
Pan-cancer clustering on common epigenetic signature clusters CIMP-positive and CIMP-negative tumors independently of tissue type. **a** Venn diagram of the CIMP signatures for each tissue. **b** Hierarchical clustering on the common epigenetic signature for all tissues. **c** Gene ontology analysis of the genes associated with the promoters of the common epigenetic signature

Comparing the epigenetic signatures that define CIMP for each tissue, we find a common set of 89 CGIs associated with 51 genes (Fig. 1a). If the signatures were random subsets of 5 % of all CGIs independent from each other, the overlap would contain on average (5 *%*)^5^≃3.10^−5^*%* of all CGIs, namely 0.006 CGI. This provides a strong evidence that a common set of genes is involved in CIMP in different cancers. We call these 89 CGIs the *cross-cancer CIMP signature* (Table 3). A hierarchical clustering on all samples restricted to this cross-cancer CIMP signature is able to cluster CIMP-positive and CIMP-negative patients independently of the tissue of origin (Fig. 1b), suggesting that CIMP observed in each individual cancer share in common a significant proportion of genes whose promoter CGIs are hypermethylated in all CIMP-positive cancers. A functional enrichment analysis of the cross-cancer CIMP signature reveals that it is significantly enriched in genes involved in cell differentiation and neuronal developmental and immune response processes (Fig. 1c).

**Table 3:** List of genes associated with the common set of CGIs that define CIMP in each tissue

	LOC339524, GSTM1, CD1D, LMX1A
	CACNA1E,NR5A2, WNT3A, GNG4
	EMX1, CTNNA2,LRRTM1, DLX1
	EVX2, HOXD13, GBX2, SYN2
	HAND2, NBLA00301, EBF1, HIST1H2BB
Epigenetic	HIST1H3C, HLA-DRB1, C6orf186, IKZF1
Signature	CDKN2A, HMX3, KNDC1, KLHL35
	HOTAIR, SLC6A15, ALX1, RFX4
	CLDN10, ADCY4, RIPK3, NID2
	OTX2, OTX2OS1, GSC, KIF26A
	GREM1, SEC14L5, HS3ST3B1, IGF2BP1
	HOOK2, NFIX, ZNF577, ZNF649
	CPXM1, CDH22, CHRNA4

### Are there 2 or 3 CIMP classes?

Several studies suggest the existence of a third class in CIMP phenotype that corresponds to an intermediate level of methylation [12, 23, 24]. While we enforced an analysis with 2 classes to define the CIMP of each sample as positive or negative in the previous section, we now examine whether the data call for a third class. Following [25], we assess the existence of an intermediate CIMP phenotype for each tissue by comparing the increase in empirical cumulative distributive distribution *Δ*(*K*) for different values of *K*=2,…,5 where *K* is the number of clusters considered for CIMP.

**Fig. 2:**
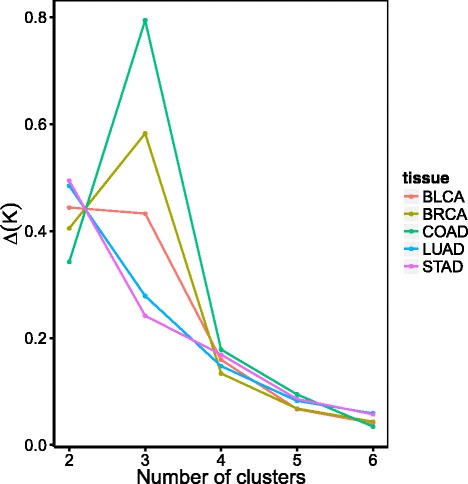
Variation in the empirical cumulative distributive function *Δ*(*K*) for each tissue. The empirical cumulative distributive function *Δ*(*K*) is a data-driven criterion which can indicate the number of clusters *K* in the data when it reaches its maximum [25]. This plots shows how *Δ*(*K*) varies as a function of *K*, for the different tissues

Figure 2 shows how *Δ*(*K*) varies as a function of *K* for each cancer, suggesting how many clusters exist in each case. We observe that the existence of a third class is not clear-cut. While colon and breast tissues show a significant increase in *Δ*(*K*) for *K*=3 suggesting a possible third cluster in CIMP, the bladder is flat between 2 and 3 clusters, while lung and gastric cancers do not support the presence of 3 classes. In addition, we assess the stability of 3 clusters by varying the number of CGIs that define CIMP and observed that while CIMP clusters are highly robust for *K*=2, there is some high variability in the cluster definitions for *K*=3 (Additional file 2). In summary, the presence of 2 clusters is well supported by the data in all cancers, while the third cluster is much more debatable.

### Similar gene expression variations are predictive of CIMP

To shed light on the relationship between methylation and transcription, we now assess to what extent a transcriptomic signature can classify the samples as CIMP positive or negative. For that purpose, we collected for each family of cancer samples with both methylation and gene expression data available, leading to a subset of samples with an overall proportion of CIMP-positive samples comparable to that of the original dataset (Table 4). We measure by cross-validation how well expression data alone can recover the two CIMP classes.

**Table 4:** CIMP Proportion in samples with both methylation and gene expression data. This table shows the number of CIMP-positive and CIMP-negative samples characterized by both methylation and gene expression data, for each cancer type, as well as the proportion of CIMP-positive samples

Tissue	Negative	Positive	Ratio (%)
Bladder	27	16	37
Breast	385	93	20
Colon	27	7	20
Lung	22	60	75
Stomach	131	178	58
Overall	*592*	*354*	*37*

**Fig. 3:**
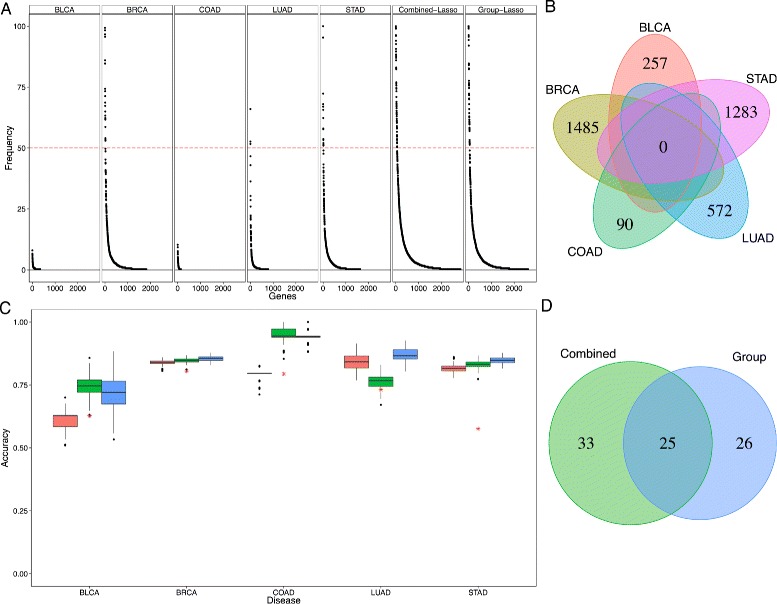
Gene expression variations predictive of CIMP. **a** Stability of each gene signature for each tissue-specific CIMP prediction as well as the “Combined-Lasso” and the “Group-Lasso” CIMP prediction task obtained and ranked by frequency of appearance using bootstrap (*n*=100 repeats). For bladder and colon CIMP prediction task, the signature was non-robust (frequency of the most redundant gene inferior to 10 *%*). The combined prediction task signature outperforms the tissue-specific signatures in robustness. **b** Venn diagram of the tissue-specific gene signatures using lasso for each tissue separately. **c** Distribution of the accuracy of the CIMP-phenotype prediction task given the patient gene expression profile using *n*=100 bootstrap and threefold cross-validation for several methods (*pink* = “tissue-specific” lasso, *green* = “Combined-Lasso,” *blue* = “Group-Lasso,” *red star* = random prediction). **d** Venn diagram representing the intersection between the “Combined” and “Group” lasso gene signatures

We first perform a multivariate regression analysis using the lasso technique to assess whether gene expression of a few genes can be predictive of the CIMP status for each tissue separately. The cross-validation accuracies for each family of cancer are shown in Table 5. We observe that while a classifier based on gene expression performs significantly better than random to recover CIMP-positive samples in breast, lung, and stomach cancers, the performance on the bladder and colon is not different from a random classifier. Moreover, we compare the lists of genes selected in the transcriptomic signature after bootstrap resampling of the samples in order to assess their robustness and potential biological significance (Fig. 3a). We observe that very few genes are robustly selected in the signatures, and in particular that no gene is associated with BLCA-CIMP and COAD-CIMP prediction in more than 15 % of the bootstrap resampling. In addition, the transcriptomic signatures of different cancers are very diverse, and no gene is present in all of them (Fig. 3b). Overall, these results suggest that there is information in the transcriptome related to the CIMP status, but that a robust signature across cancers is difficult to obtain.

**Table 5:** Accuracy of CIMP prediction using gene expression profiles

	Accuracy			
	Random	Lasso	Combined lasso	Group lasso
Bladder	62.8	62.9 (*p* = 1)	74.2 (*p*≤2.10^−16^)	72.1 (*p*≤2.10^−16^)
Breast	80.5	83.9 (*p*≤2.10^−16^)	84.7 (*p*≤2.10^−16^)	85.5 (*p*≤2.10^−16^)
Colon	79.4	79.5 (*p* = 1)	95.0 (*p*≤2.10^−16^)	94.2 (*p*≤2.10^−16^)
Lung	73.2	84.2 (*p*≤2.10^−16^)	76.2 (*p*≤2.10^−16^)	86.6 (*p*≤2.10^−16^)
Stomach	57.6	81.2 (*p*≤2.10^−16^)	83.0 (*p*≤2.10^−16^)	84.8 (*p*≤2.10^−16^)
Overall	*71.9*	*82.4*	*82.6*	*85.0*

This table shows the accuracy, assessed by threefold cross-validation repeated 100 times over each tissue (first column), of sample classification in CIMP-positive and CIMP-negative classes from gene expression data using random classification (second column), lasso logistic regression (third column), combined lasso (fourth column), or group lasso logistic regression (fifth column)

However, the poor accuracy as well as the non-robustness of genetic signatures to predict CIMP may be due to the small size of some datasets (*n*_BLCA_=43, *n*_COAD_=34). To overcome the lack of statistical power due to small sample size, we combine in a second analysis the different datasets into a single multivariate regression analysis, based on the assumption that the CIMP signatures of different cancers may share the same genes. We train classifiers to predict CIMP status from gene expression data jointly across cancers using two methods, based on two different assumptions: (i) assuming that all tissues share the same gene signature and coefficients for the prediction task, we run a single lasso classification on the combined datasets (“Combined-Lasso” prediction) or (ii) assuming that all tissues share the same gene signature but with different coefficients, we jointly train several models with a group lasso approach to constrain the selected genes to be the same across cancers without imposing their coefficients to coincide (“Group-Lasso” prediction) (see supplementary methods in Additional file 3). The rationale for the group lasso approach is that while CIMP may be caused by a common subset of genes, the specific contribution of each gene may vary between tissues. Our results show that both methods significantly outperform the tissue-specific predictions (*P*≤2.10^−16^, Fig. 3c, Table 5) in particular for the bladder and colon where the size of the initial datasets could not give sufficient statistical power to predict CIMP accurately. There is overall little difference between both methods, with the notable exception of lung cancer where the combined lasso approach is significantly worse than the group lasso (and even the single lasso) model, suggesting that in that case, the weights of the genes in the CIMP signature may differ from other cancers. More importantly, each method allows to identify a common genetic signature (51 genes for the “Combined” prediction and 58 genes for the “Group-Lasso” prediction) that distinguishes CIMP-positive and CIMP-negative class for each tumors which is more robust than all the tissue-specific signatures (Fig. 3d). In addition, these signatures share a large common set of genes (25 common genes, Table 6). We represented the gene expression distribution for this common set of genes on the different datasets and observe a clear separation between CIMP-positive and CIMP-negative classes for all tissues (Additional file 4). Gene ontology analysis on the intersection of the two predictive gene signatures showed specific enrichment only for genetic regulatory processes.

**Table 6:** Intersection of the genetic signatures for “Combined-Lasso” and “Group-Lasso” predictive of CIMP ranked by decreasing level of robustness

	*ZIC2*, *AMH*, *LHX1*,
	*ZIC3*, *XKR9*,*TNNT1*,
Over-expressed	*CAMK2N2*,*PCDHB9*, *RAET1K*,
	*HIST1H2AB*, *C2CD4C*, *FBXL20*,
	*TFCP2L1*
	*MAGEC2*, *ZNF300*,*SLC15A1*,*TSPYL5*,
	*MLF1*, *GATA2*, *MAGEA12*,
Under-expressed	*LOC441666*, *MAGEA2*, *LOC389493*, *H2AFY2*,
	*LDHC*

### A genetic signature is associated to CIMP only for colon and gastric cancers

Several somatic mutations have been found to be tightly associated with epigenetic aberrations in CIMP. Recent studies have pointed out the causal role of IDH1 mutations in Glioblastoma-CIMP [17, 19] and tight associations between IDH2 and TET2 mutations with other CIMPs (leukemia [26], enchondroma, and spindle cell hemangioma [27, 28]). In the colon, BRAF and KRAS mutations are associated with microsatellite instability and COAD-CIMP [9].

**Fig. 4:**
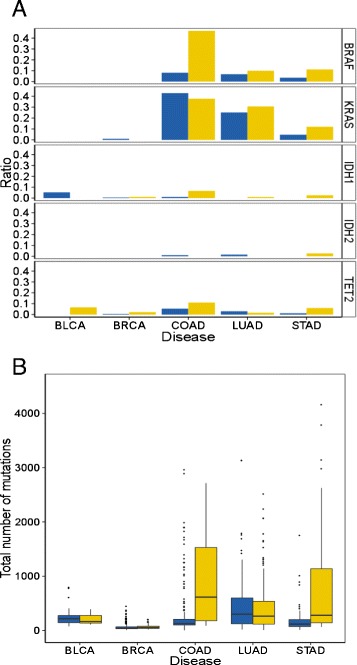
Mutation analysis. **a** Association between specific mutations (*IDH1*, *IDH2*, *BRAF*, and *KRAS*) with the CIMP phenotype for all tissues (*yellow* = CIMP positive, *blue* = CIMP negative). **b** Significantly higher mutation rate for CIMP-positive (*yellow*) compared to CIMP-negative (*blue*) tumors is observed for colon and gastric cancers only and is concordant with CIMP association with microsatellite instability for these tissues

We re-assess the association between mutations in these genes and CIMP in the different types of cancers (Fig. 4a). We recover a strong association between BRAF mutation and CIMP-positive colon tumors but no specific association with other tumor types. We also find no coordinated association between *IDH1*, *IDH2*, *KRAS*, *BRAF*, or *TET2* mutations and CIMP phenotypes for all tissues. In addition, we perform genome-wide mutation analysis to assess whether specific gene mutations are associated with CIMP. We find no significant gene mutation association for bladder, breast nor lung CIMPs. For colon and gastric cancer, we find respectively 459 and 1070 gene mutations associated with CIMP with a common intersection of 195 genes (Additional file 5 panel A). Gene ontology analysis of this set of genes shows significant enrichment for extracellular matrix organization and cell adhesion but also neuronal developmental processes (Additional file 5 panel B).

Finally, we also look at the rate of mutations in each tissue given the CIMP phenotype. We observe a significant association between the number of mutations and the CIMP status for colon and gastric cancer (Fig. 4b), in accordance with the tight association between CIMP and microsatellite instability for these two tissues [9, 29–31]. However, the same observation could not be made for the bladder, breast, and lung.

### Clinical impact of CIMP

Survival analysis in several CIMP studies has often shown distinct outcome between CIMP-positive and CIMP-negative tumors. However, there is no consensus in the general survival associated with CIMP: while CIMP has been associated with improved survival and lower risk of metastasis in breast [14], colorectal [9], leukemia [32–35], or gliomas [17], it has also been reportedly associated with poor survival for bladder [36], lung [15, 37], or prostate cancers [38], and prognosis even remains unclear for gastric cancers [39–43].

We perform a systematic survival analysis on the different tissues to assess the clinical impact of CIMP. However, we observe no significant association between CIMP and survival, in any of the tissues (Table 7 and Additional file 6).

**Table 7:** Clinical impact of CIMP. Overall survival proportion given the CIMP phenotype and the *p* value associated with the survival analysis (logrank test)

Tissue	Event		*p* value
	CIMP −	CIMP +	
BLCA	47/214	21/96	0.74
BRCA	29/495	9/114	0.20
COAD	28/218	6/54	0.57
LUAD	24/127	67/295	0.49
STAD	26/141	20/193	0.29

Other clinical parameters have been associated with CIMP such as microsatellite instability (MSI) in the colon [9] and hormone receptor statuses in the breast [14]. We therefore assess the association between the CIMP status and eight clinical annotations provided in the TCGA, namely, age, MSI, ER status, PR status, HER2 status, tumor size, lymph node invasion, and presence of metastasis. We first observe that CIMP is significantly associated with a higher age in the breast, colon, and stomach (*P*_breast_=2.10^−4^, *P*_colon_=2.10^−3^, *P*_stomach_=0.036, student test, Additional file 7 panel A) but not in the bladder and lung. In the colon, we recover a significant association between CIMP and MSI (*P*=5.10^−6^, chi-squared test, Additional file 7 panel B). We also recover a significant association between CIMP and ER, PR, and HER2 statuses in breast (*P*_ER_=2.10^−5^, *P*_PR_=0.03, *P*_HER2_=5.10^−8^, chi-squared test, Additional file 7 panel C). However, we observed no significant association between CIMP and either tumor size, lymph node invasion, or metastasis in any tissue.

## Discussion

CIMP has been thoroughly studied over the past few years in several tissue types but the heterogeneity of the methods and measurement technologies has hindered the assessment of a common epigenetic and genetic signature predictive of CIMP across all cancer sites [22]. In the present study, we analyze a large dataset of over 2000 tumor methylation profiles measured with a single technology from 5 different tissue types. We observe a universal epigenetic signature that defines CIMP independently from the tissue of origin, which might suggest a common molecular basis to CIMP across tissues. Genes associated with these CGIs are enriched in several biological pathways linked to organ development and include several interesting genes such as *CDKN2A* coding for p16, a well-characterized tumor suppressor protein [44], which is aberrantly hypermethylated in CIMP-positive tumors and might contribute to tumor development. Other genes present in the cross-cancer CIMP signature such as *HOTAIR*, which is known to reprogram the chromatin state and is associated with breast cancer metastasis [45], might on the contrary be repressed in CIMP tumors and be linked with a better prognosis for breast cancer patients. *GREM1* is another gene present in the CIMP signature and is associated with tumor cell proliferation [46]. Less documented genes present in the CIMP signature could potentially be investigated for a biological validation of their role in tumor development.

Recent studies have pointed out that epigenetic aberrations could be derived from genetic aberrations [47]. By combining the different datasets into a single prediction task, we are able to identify a common set of genes whose expression levels can predict the CIMP status for each tissue. This gene list is enriched mostly in genetic regulatory pathways, suggesting that the epigenetic reprogramming and thus CIMP might be an intermediate step in the regulatory mechanism. Among the genes contained in the signature, *ZIC2*, which is robustly selected in each bootstrap of the CIMP prediction task and is significantly more expressed in CIMP-positive tumors for each tissue, has been known to act as a Wnt/ *β*-catenin signalling inhibitor [48] which is usually upregulated in several cancers. Another interesting characteristic of this genetic predictive signature from a clinical point of view is the recurrence of cancer/testis antigens (CTAs) such as *MAGEC2* [49–51], *MAGEA12* [52, 53], *MAGEA2* [54], and *LDHC* [55], which are interesting targets for cancer immunotherapy [56] and are consistently under-expressed in CIMP-positive tumors. Recently, Gevaert et al. [57] also showed a strong association between *MAGEA4* hypomethylation and CIMP-positive tumors which further supports the link between CTAs and the absence of a methylator phenotype.

Mutation analyses are not very conclusive in defining a set of specific somatic mutations significantly associated with CIMP. In particular, lowly mutated cancer sites such as the bladder, breast, or even lung do not show any mutations significantly associated with CIMP. For highly mutated cancer sites such as colon or stomach, our results confirm a strong association between *BRAF* mutation and COAD-CIMP [9] but do not show any particular associations with *IDH1/2*, which have been reported to be causal in gliomas and leukemia [19, 26]. There is a strong association between COAD and STAD-CIMP and the specific mutations of genes related with extracellular matrix and cell adhesion, both reported to be strongly associated with metastasis [58–61]. Interestingly, neuronal developmental processes are highly enriched but affecting different genes from the universal epigenetic signature. Associations with neuronal development were already mentioned in [17].

Studies have often reported a clear distinct clinical prognosis associated with CIMP [9, 14, 17, 32]. This reiterates that a main reason for defining CIMP in each tissue site is its potential use as a prognosis marker. However, CIMP could be associated with a good or bad prognosis depending on the type of tumors. In the current study, we do not observe a significant association with any good nor bad prognosis linked with CIMP.

## Conclusion

This meta-analysis of more than 2000 samples sheds new light on CIMP across cancers, its link with gene expression, and its clinical relevance. We found strong evidence that a panel of genes, which we call the pan-cancer CIMP signature, is involved simultaneously in the establishment of the CIMP in various cancer sites, which might be an indicator of a universal biological process behind CIMP. We found that differences in the CIMP status of a sample is associated to differences in the transcriptome, and also found a core set of genes whose expression levels differentiates CIMP-positive and CIMP-negative samples, in all cancers studied. Finally, we found little evidence of association between CIMP and mutations, except for the well-known BRAF mutation in colon cancer and also little association with patient survival.

## Materials and methods

### Patient selection

All data were retrieved from the TCGA data portal. We selected samples from bladder, breast, colon, lung and gastric adenocarcinomas because large matched datasets were available for methylation, gene expression, and mutation profiles. Moreover, all these tissues were previously reported to exhibit a methylator phenotype. The datasets are detailed in Table 1 and the different institutions that released the data are mentioned in the “Acknowledgements” section.

### Methylation profiling

Methylation profiles were retrieved from level 2 TCGA data. They were obtained with the Illumina HumanMethylation450K DNA Analysis BeadChip assay, which is based on genotyping of bisulfite-converted genomic DNA at individual CpG sites to provide a quantitative measure of DNA methylation [62]. Following hybridization, the methylation value for a specific probe was calculated as the ratio *M*/(*M*+*U*) where *M* is the methylated signal intensity and *U* is the unmethylated signal intensity. Across the genome, 485,577 CpG methylation levels, associated with 27,176 CGIs and 21,231 genes, were measured as such.

Following [63], we considered not only the CGI methylation profile but also included in the analysis proximal regions in the near vicinity (up to 4 kb), namely the CGI Shores and Shelves regions in a general CGI + SS methylation profile.

### Gene expression profiling

Gene expression profiles were retrieved from level 3 TCGA data. They were obtained from the Illumina HiSeq RNASeq technology and processed following [64]. We used the reads per kilobase per million mapped reads (RPKM) to quantify the gene expression level from RNA sequencing data.

### Mutation profiling

Mutation profiles were retrieved from somatic mutation profiles from level 2 TCGA data obtained through whole exome sequencing. To compare the rate of mutation given the CIMP status, we performed a hypergeometric test and corrected for multiple testing using Benjamini-Hochberg correction.

### CIMP analysis

To assess the existence of CIMP, we performed Ward hierarchical clustering using euclidean distance on the top 5 % most variant CGIs. Variations from 1 to 10 % of the most variant CGIs had a small impact on the clustering stability (Additional file 3). We then cut the hierarchical clustering tree in two classes namely CIMP-positive and CIMP-negative tumors given their average level of methylation (CIMP-positive = high level of methylation, CIMP-negative = low level of methylation). Robustness of the clustering was obtained through consensus clustering [25].

### Predicting CIMP status from gene expression profiles

We performed logistic regression using a lasso penalty [65] to predict CIMP status from gene expression profiles for each tissue separately. Accuracy is calculated through threefold cross-validation averaged over 100 repeats. To combine the different datasets into a single prediction task, we performed group-lasso logistic regression (Additional file 1). Given the imbalanced proportion of CIMP in each datasets, we defined the “random” predictor as a predictor that always predicts the majority class. The statistical significance of a gene expression-based predictor over the “random” predictor was calculated using a Student *t* test.

To determine the genetic predictive signature, genes were ranked according to the frequency at which they appeared in the optimal lasso estimator signature averaged over the different folds and repeats. Genes with a frequency of at least 50 % were selected.

### Survival analysis

Overall survival was estimated using the Kaplan-Meier method [66] to compare the survival between CIMP-positive and CIMP-negative tumors. A multivariate Cox proportional hazards regression model [67] was also fitted to assess the CIMP odd ratio.

## Endnotes

^1^Bladder tissue ^2^Breast tissue ^3^Colon tissue ^4^Lung tissue ^5^Stomach tissue

## Additional files

Additional file 1
**Hierarchical clustering and CIMP status of samples in each tissue.** Each sample is represented by the methylation levels of the 5 % of the probes that vary most in the tissue considered. Heatmaps range from hypomethylated (*blue*) to hypermethylated (*yellow*). The *column colorbar* represents the resulting assignment of each sample as CIMP positive (*yellow*) or CIMP negative (*blue*). Panel A. bladder; panel B. breast; panel C. colon; panel D. lung; panel E. stomach. (PDF 13722 kb)

Additional file 2
**Stability of CIMP clusters with respect to the size of the CIMP signature.** Robustness of cluster assignment for each sample (*columns*) as a function of the proportion of variant CGIs kept to define the CIMP signature, from 1 to 10 % (*rows*) and given the number of CIMP clusters considered (*left panels*: K = 2, *right panels*: K = 3, *yellow* = CIMP-positive, *blue* = CIMP-negative, *black* = CIMP-low) for bladder (panel A/B), breast (panel C/D), colon (panel E/F), lung (panel G/H), stomach (panel I/J). Panel K. Table summarizing the stability of the cluster assignments for each tissue and different number of CIMP clusters considered. (PDF 381 kb)

Additional file 3
**Supplementary methods.** (PDF 153 kb)

Additional file 4
**Gene expression profiling on the common genetic predictive signature for each tissue.** The *column color bar* represents the CIMP status (*yellow* = CIMP-positive, *blue* = CIMP-negative) while the *row color bar* represents the clustering of genes (*green* = under-expressed in CIMP, red = over-expressed in CIMP). Panel A. bladder; panel B. breast; panel C. colon; panel D. lung; panel E. stomach. (PDF 561 kb)

Additional file 5
**Study of a genetic signature associated with CIMP.** Panel A. Venn diagram representing the intersection of the mutations significantly associated with CIMP in colon and gastric cancers. Panel B. Gene ontology analysis of the common genes associated with CIMP. (PDF 82 kb)

Additional file 6
**Clinical impact of CIMP on the patient surival.** The plots show the Kaplan Meier survival curves based on CIMP status for different tissues. Panel A. bladder; panel B. breast; panel C. colon; panel D. lung; panel E. stomach. (PDF 89 kb)

Additional file 7
**Association between CIMP and clinical annotations.** Panel A. Association between CIMP and age: distribution of patients’ age given their CIMP phenotype in each tissue. Panel B. Association between CIMP and MSI in colon: ratio of MSI-positive and MSI-negative patient given the CIMP phenotype in the colon. Panel C. Association between CIMP and ER status in breast: ratio of ER-positive patients given the CIMP phenotype in the breast. Panel D. Association between CIMP and PR status in the breast: ratio of PR-positive patients given the CIMP phenotype in the breast. Panel E. Association between CIMP and HER2 status in the breast: ratio of HER2-positive patients given the CIMP phenotype in the breast. (PDF 86 kb)

## Abbreviations

CIMP: CpG island methylator phenotype
CGI: CpG island
BLCA: Bladder carcinoma
BRCA: Breast carcinoma
COAD: Colon adenocarcinoma
LUAD: Lung adenocarcinoma
STAD: Stomach adenocarcinoma
