# Supplementary figures and images for: Integrative DNA methylation and gene expression analysis to assess the universality of the CpG island methylator phenotype
Source: Hum Genomics. 2015 Oct 13;9:26. doi: 10.1186/s40246-015-0048-9 (PMC4603341; doi:10.1186/s40246-015-0048-9)

A.

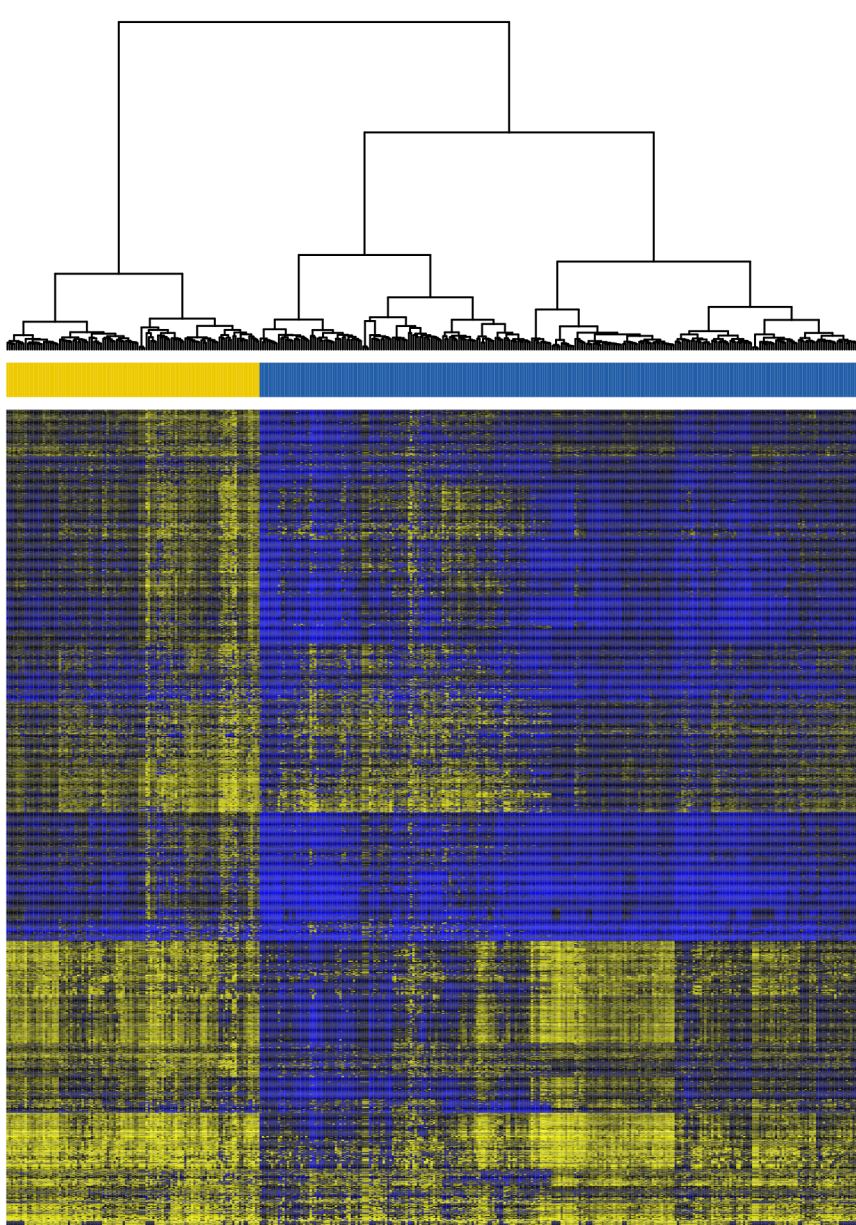

B.

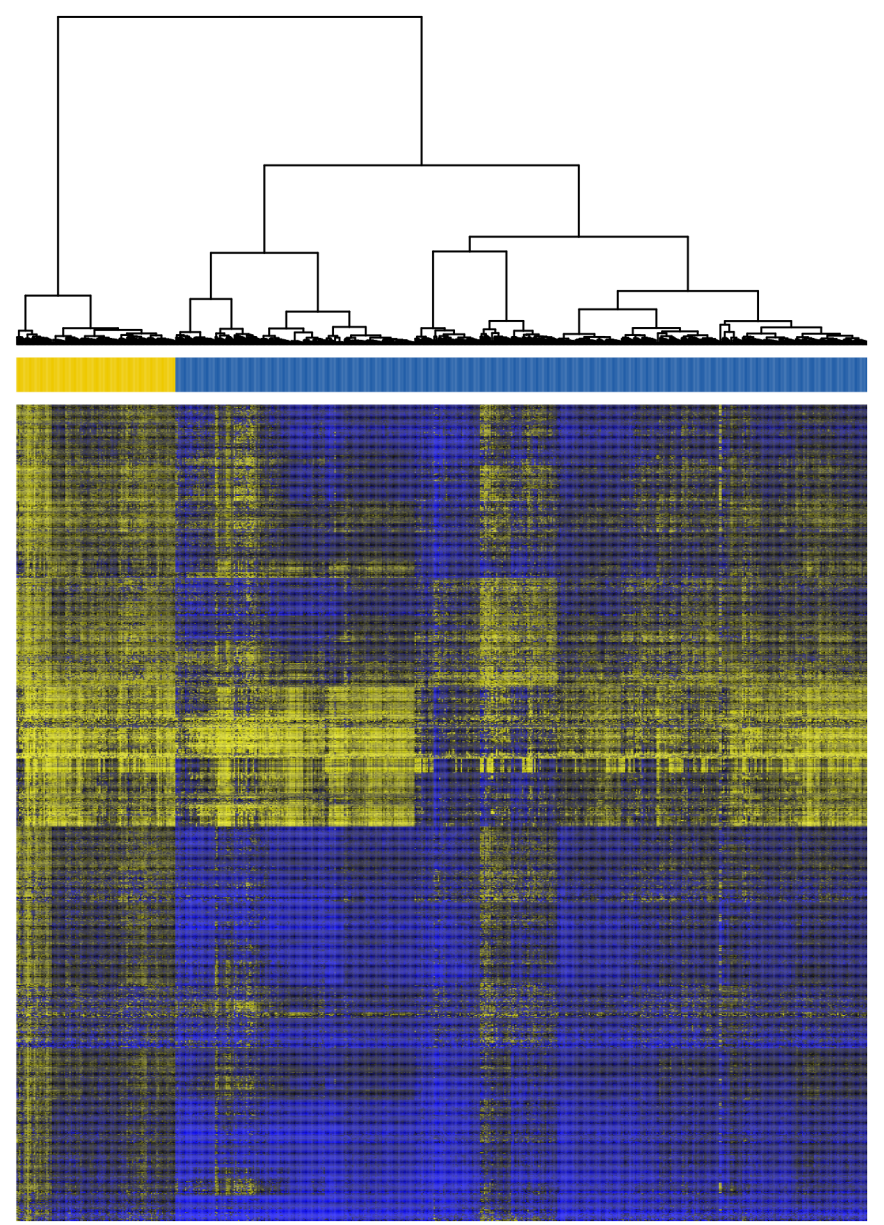

C.

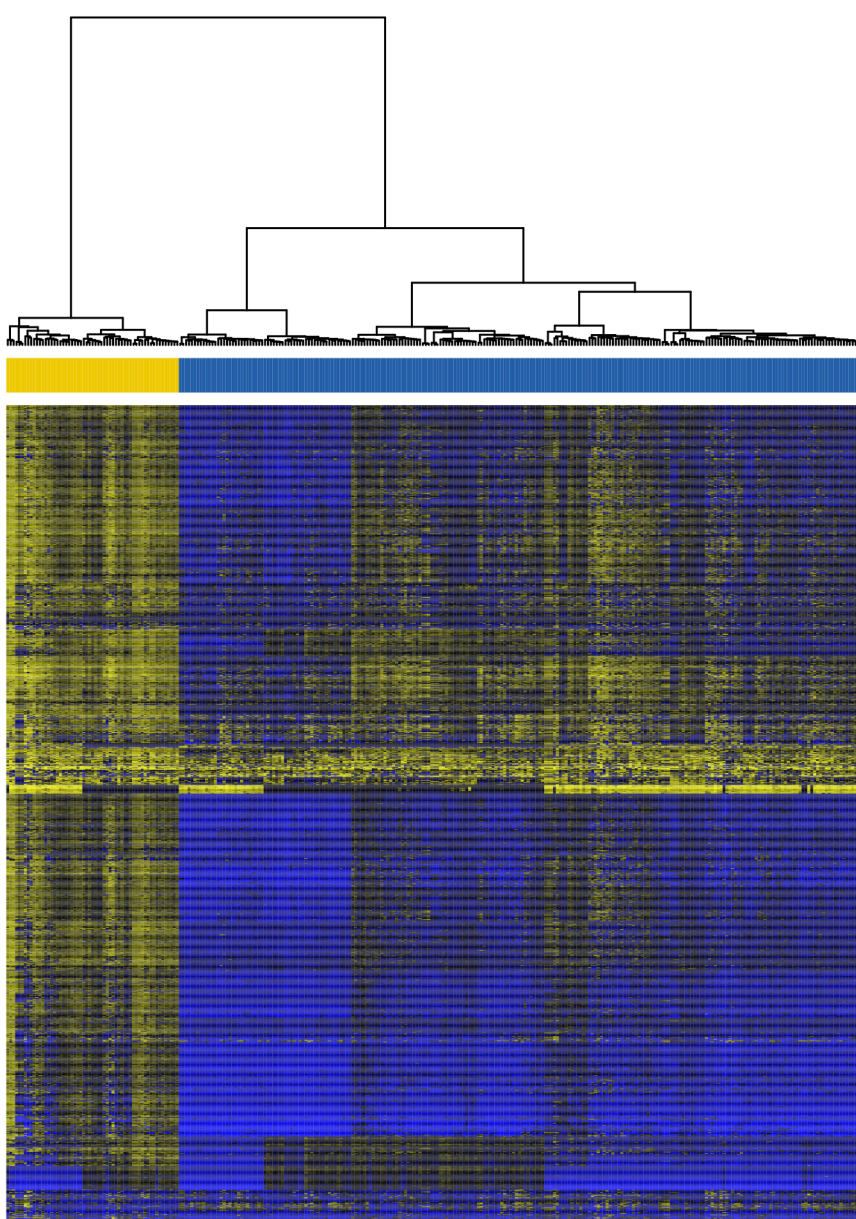

D.

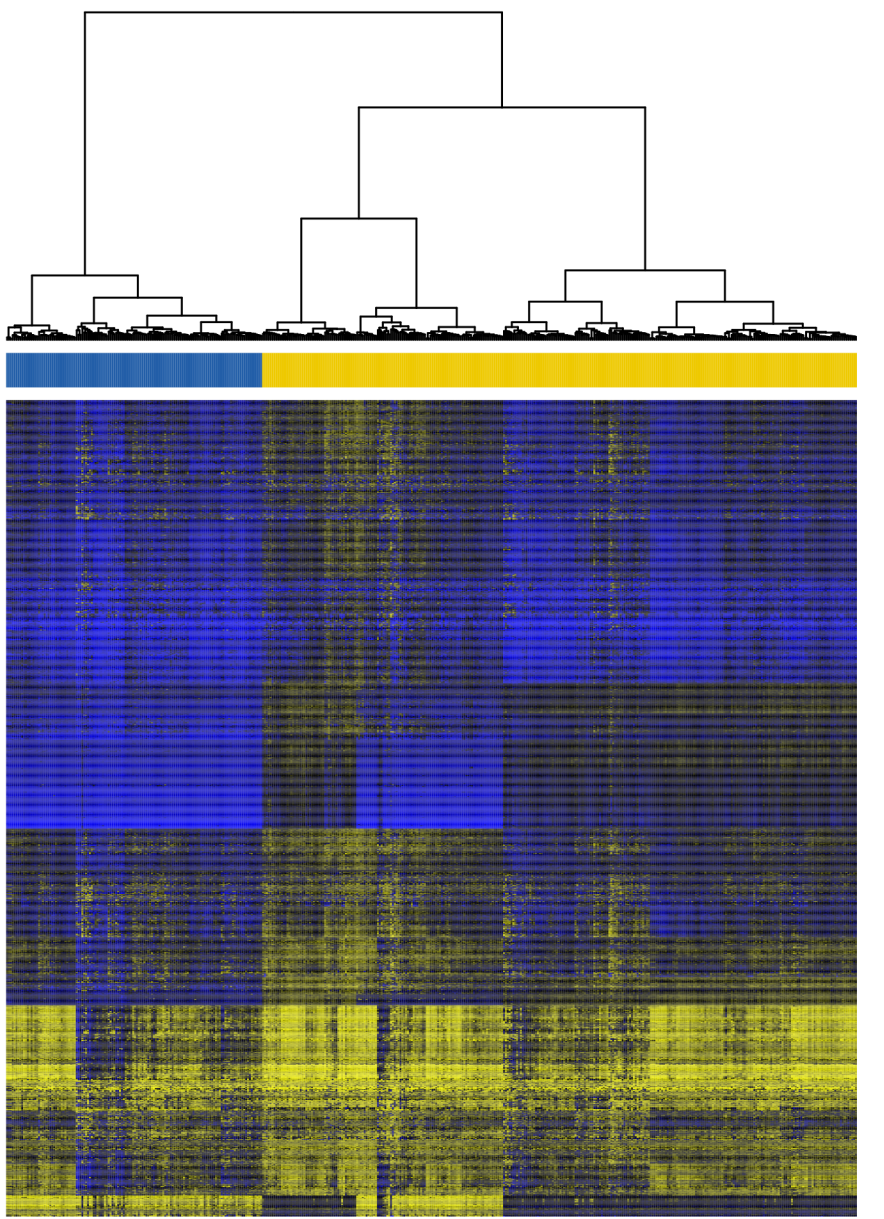

E.

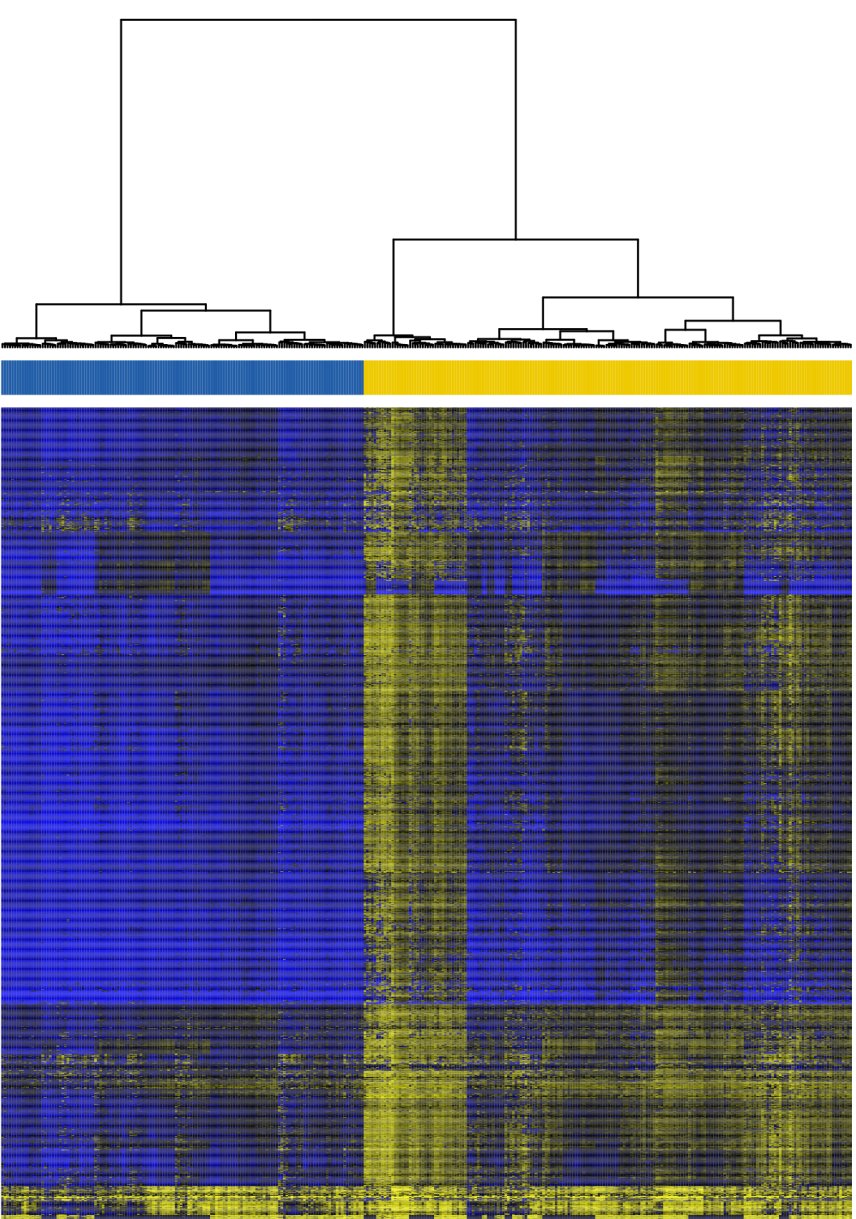

Supplement: Additional file 1 — Hierarchical clustering and CIMP status of samples in each tissue. Each sample is represented by the methylation levels of the 5 % of the probes that vary most in the tissue considered. Heatmaps range from hypomethylated (blue) to hypermethylated (yellow). The column colorbar represents the resulting assignment of each sample as CIMP positive (yellow) or CIMP negative (blue). Panel A. bladder; panel B. breast; panel C. colon; panel D. lung; panel E. stomach. (PDF 13722 kb) [file 40246_2015_48_MOESM1_ESM.pdf]

A.

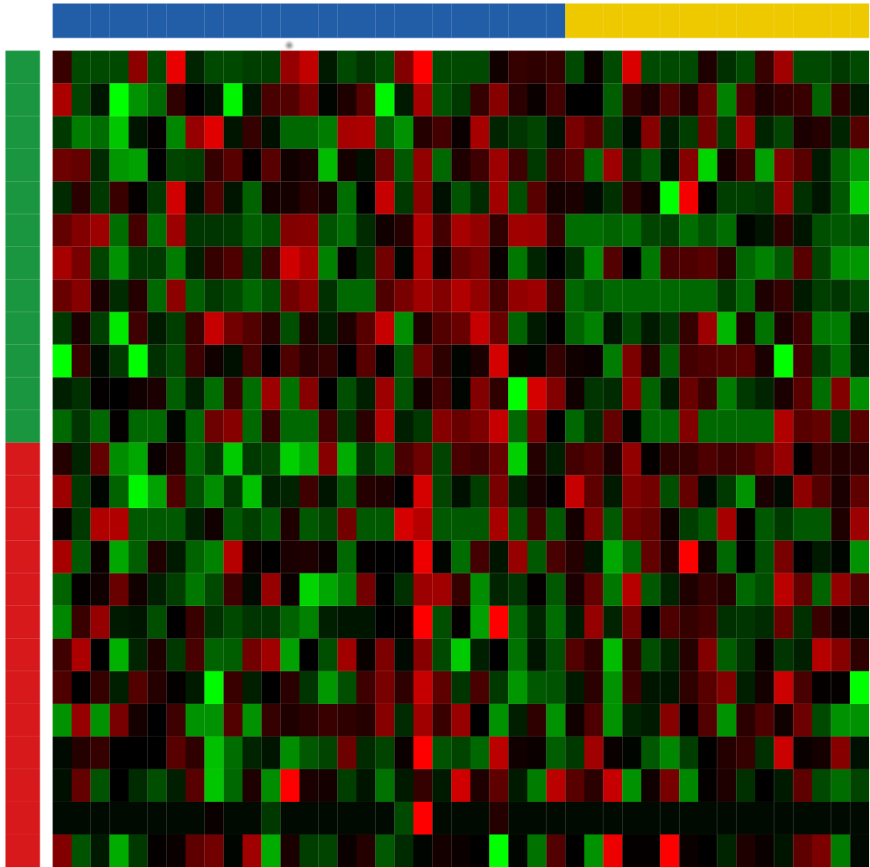

B.

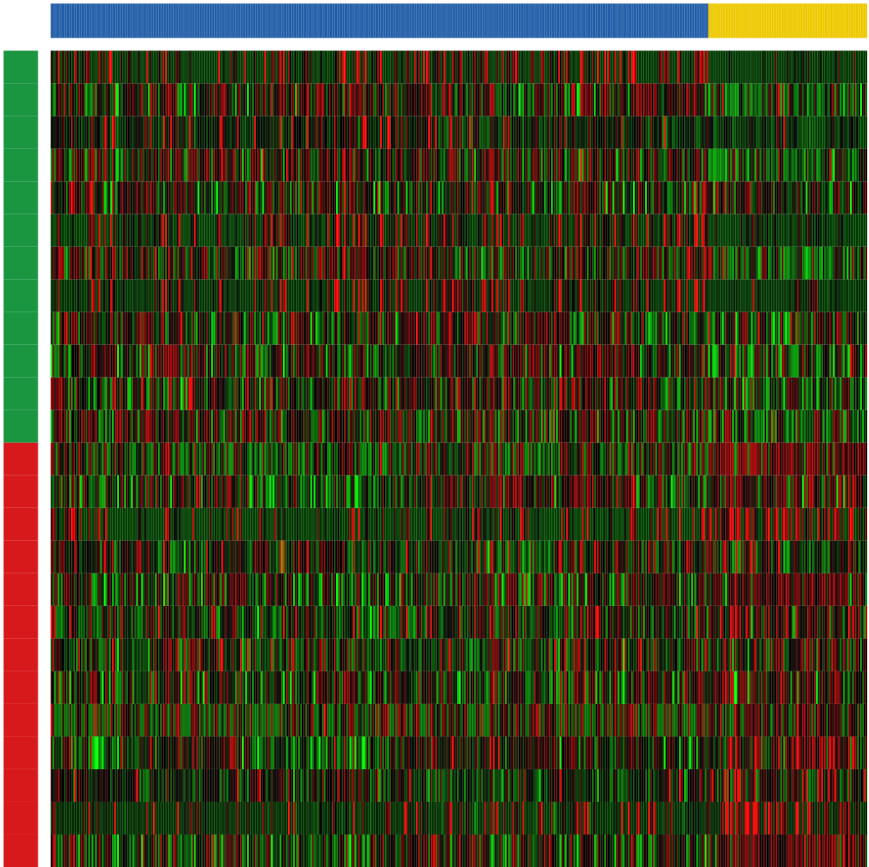

C.

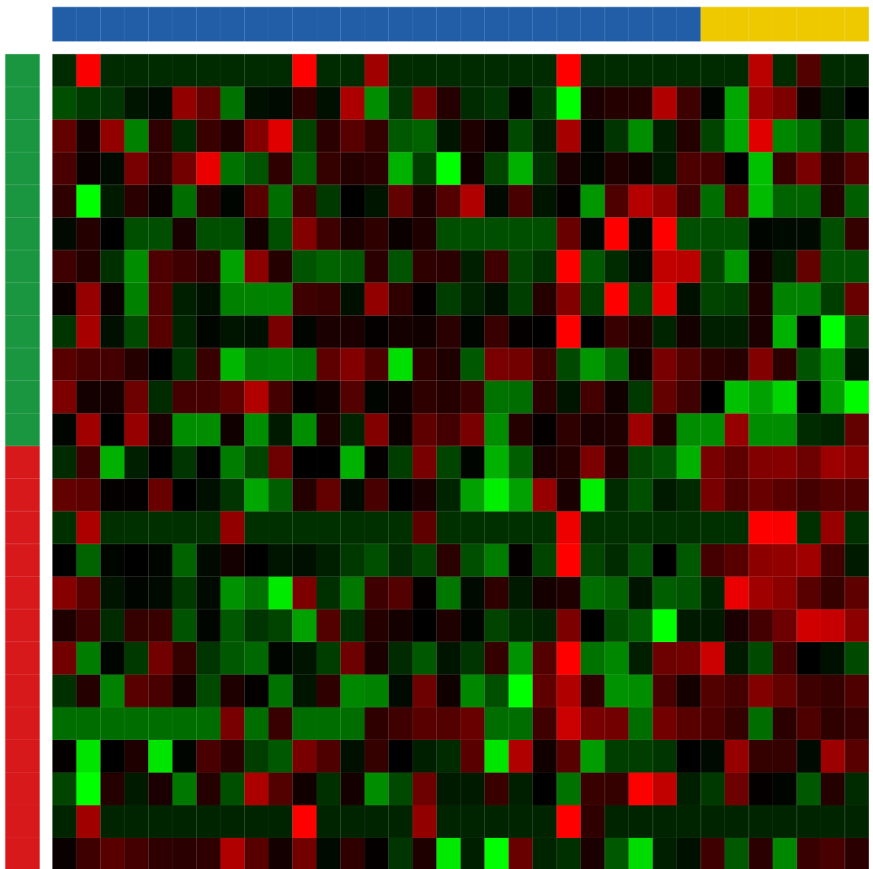

D.

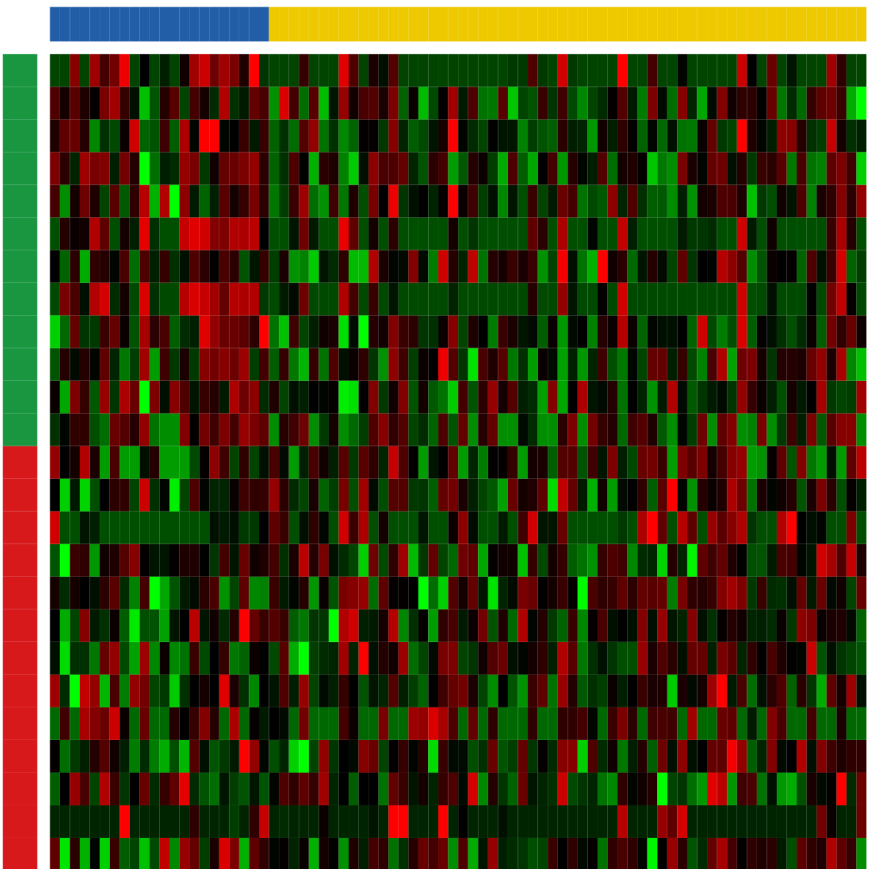

E.

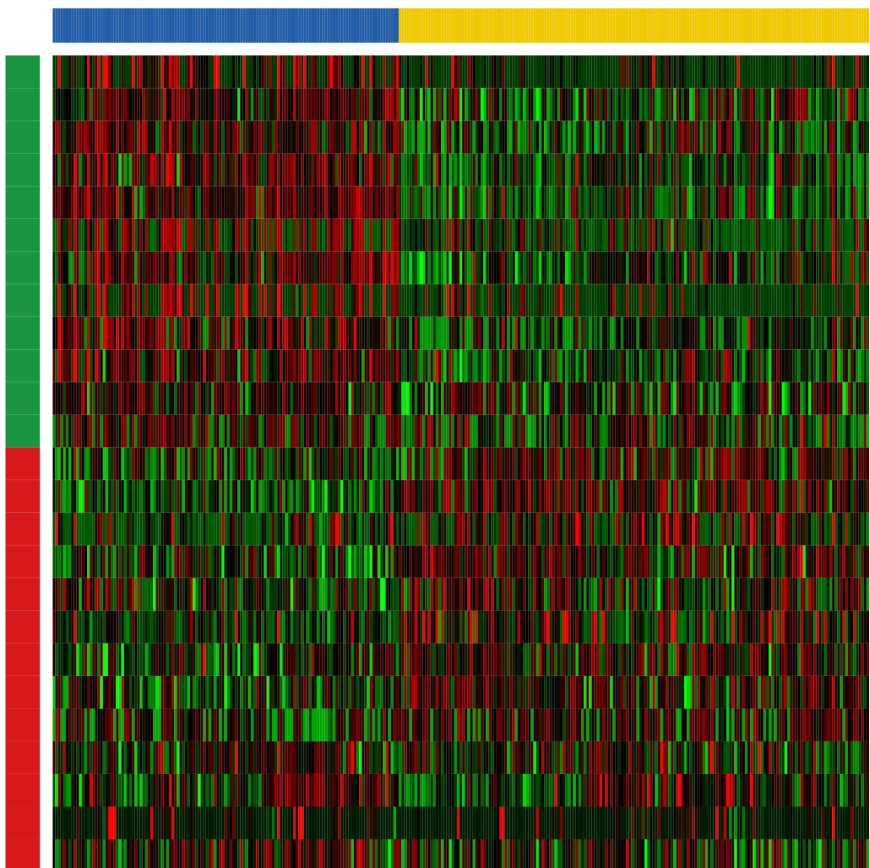

Supplement: Additional file 4 — Gene expression profiling on the common genetic predictive signature for each tissue. The column color bar represents the CIMP status (yellow = CIMP-positive, blue = CIMP-negative) while the row color bar represents the clustering of genes (green = under-expressed in CIMP, red = over-expressed in CIMP). Panel A. bladder; panel B. breast; panel C. colon; panel D. lung; panel E. stomach. (PDF 561 kb) [file 40246_2015_48_MOESM4_ESM.pdf]

A.

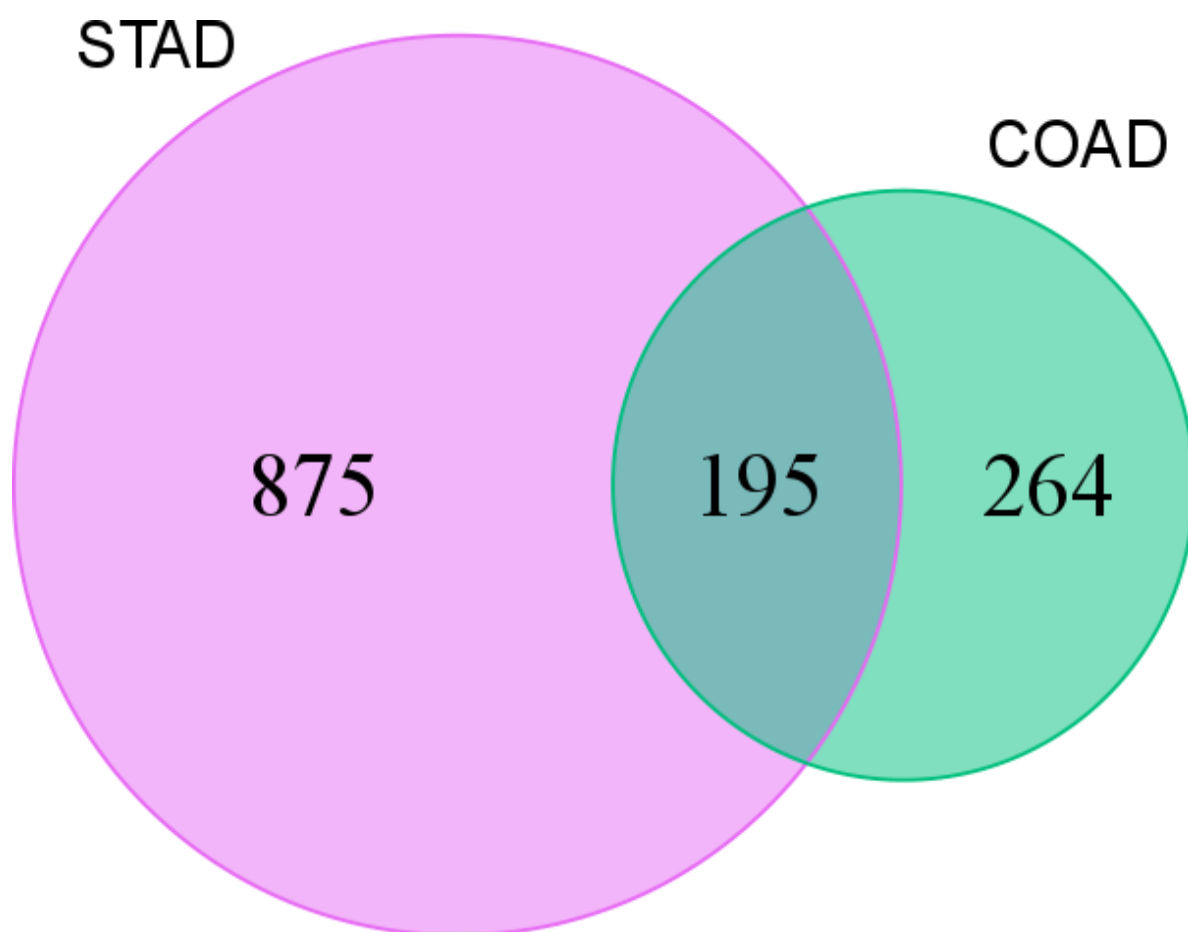

B.

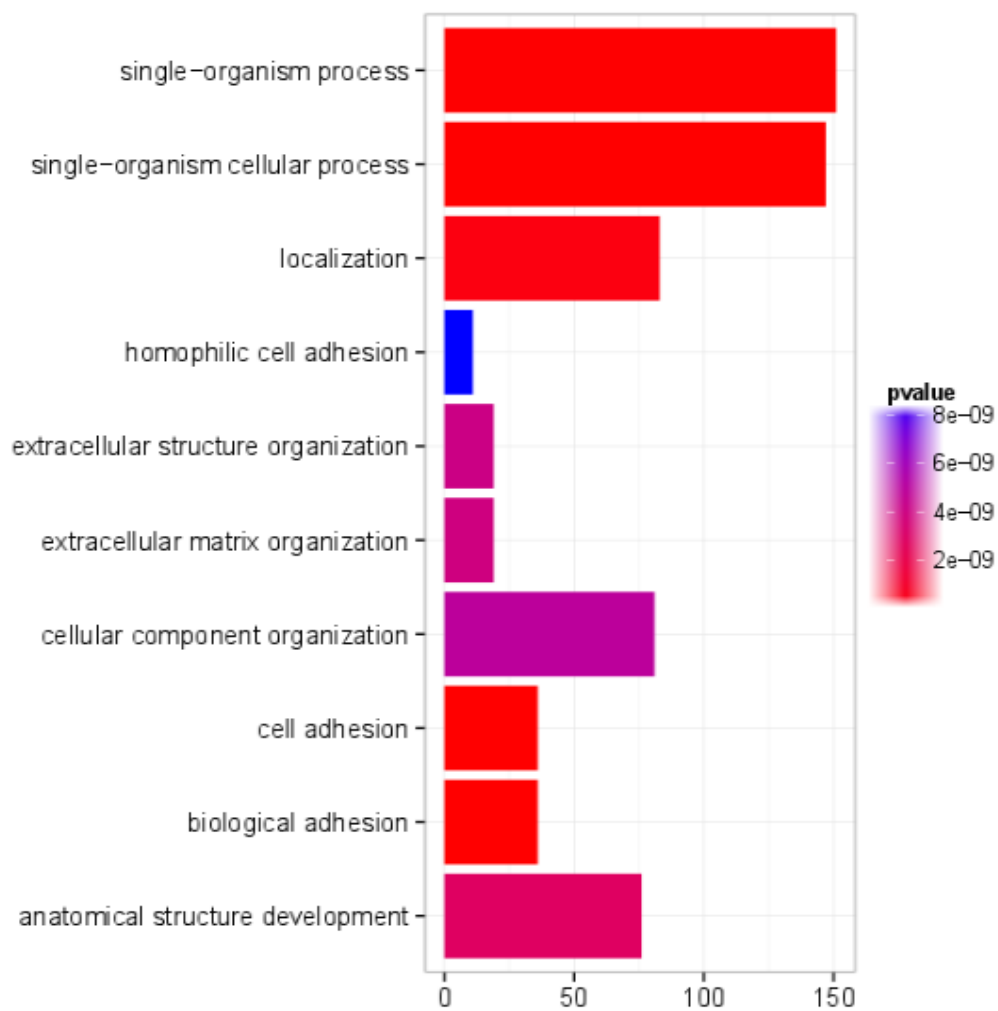

Supplement: Additional file 5 — Study of a genetic signature associated with CIMP. Panel A. Venn diagram representing the intersection of the mutations significantly associated with CIMP in colon and gastric cancers. Panel B. Gene ontology analysis of the common genes associated with CIMP. (PDF 82 kb) [file 40246_2015_48_MOESM5_ESM.pdf]

**A.**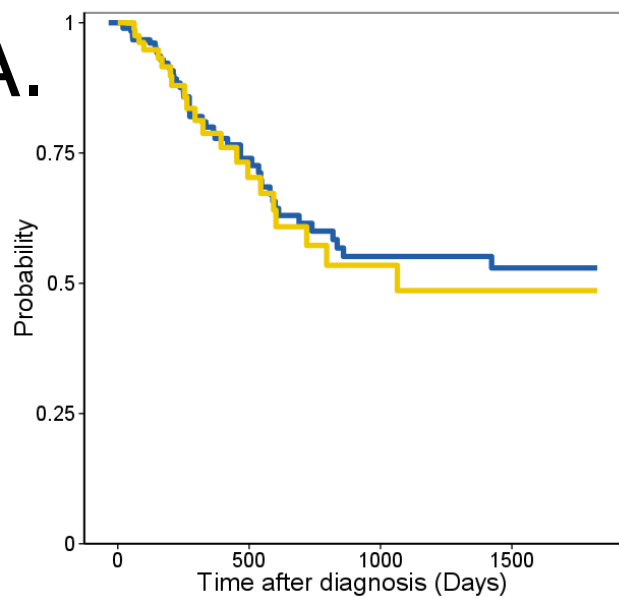**B.**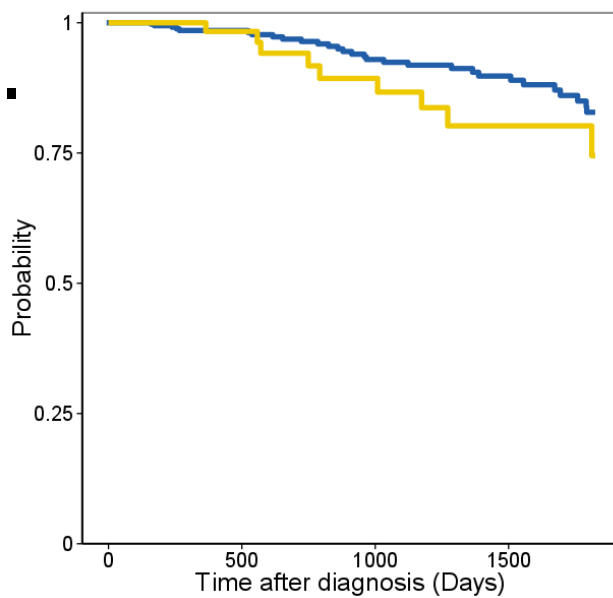**C.**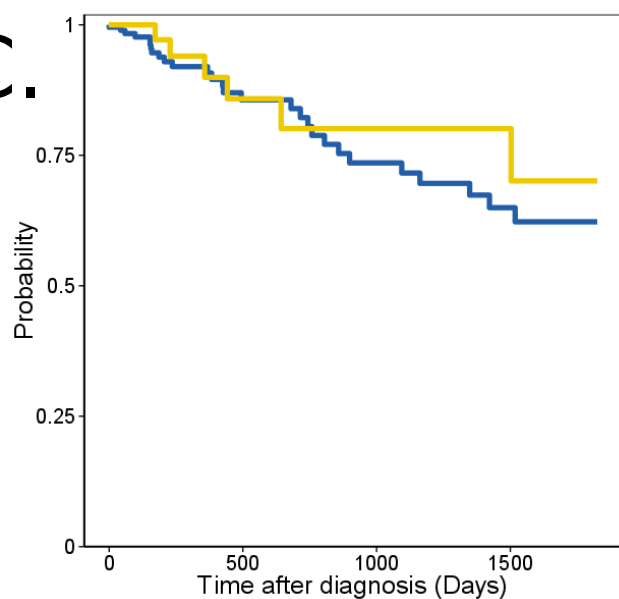**D.**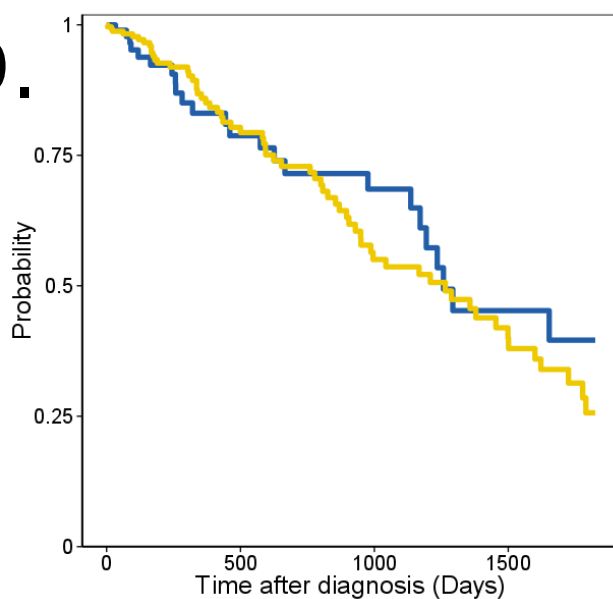**E.**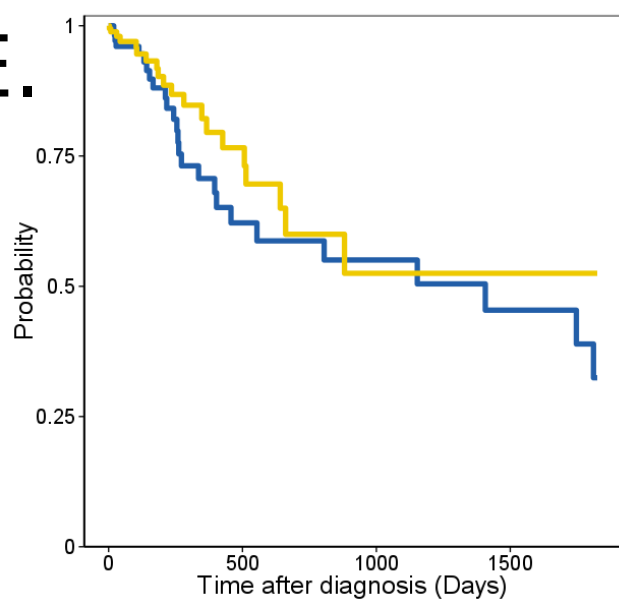

Supplement: Additional file 6 — Clinical impact of CIMP on the patient surival. The plots show the Kaplan Meier survival curves based on CIMP status for different tissues. Panel A. bladder; panel B. breast; panel C. colon; panel D. lung; panel E. stomach. (PDF 89 kb) [file 40246_2015_48_MOESM6_ESM.pdf]

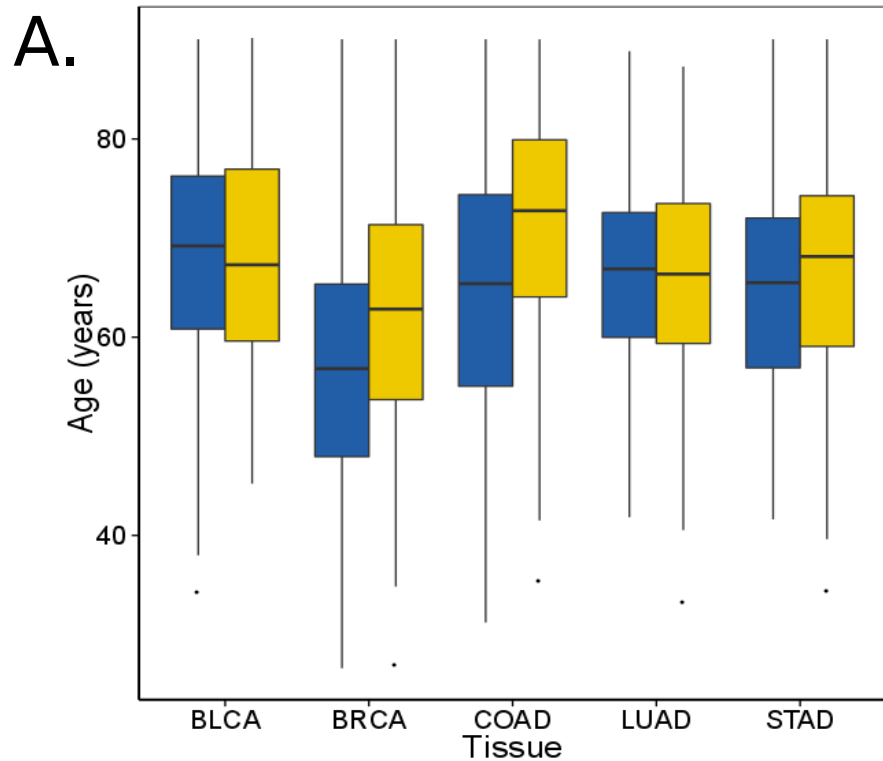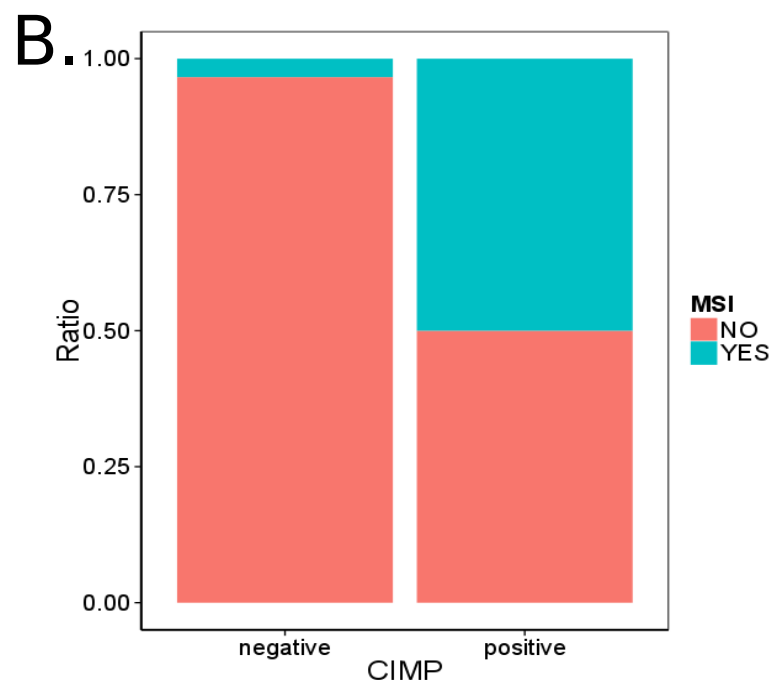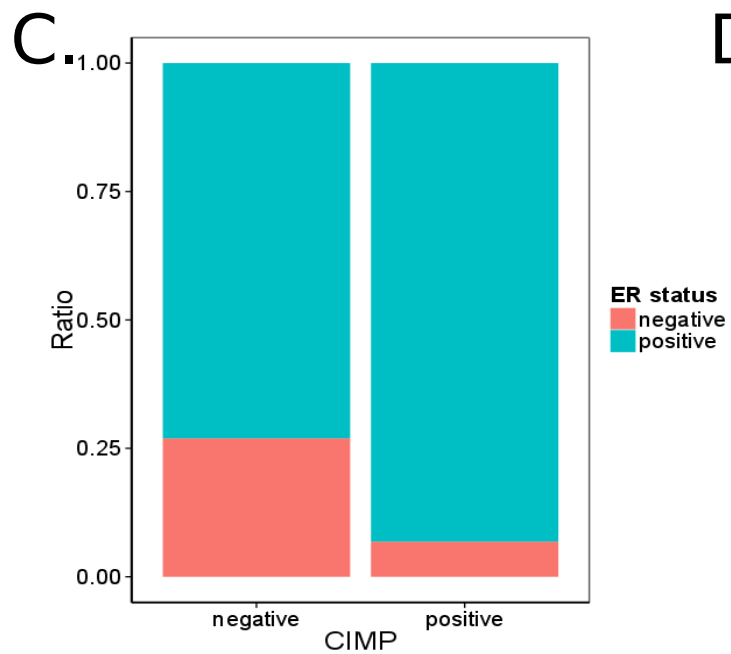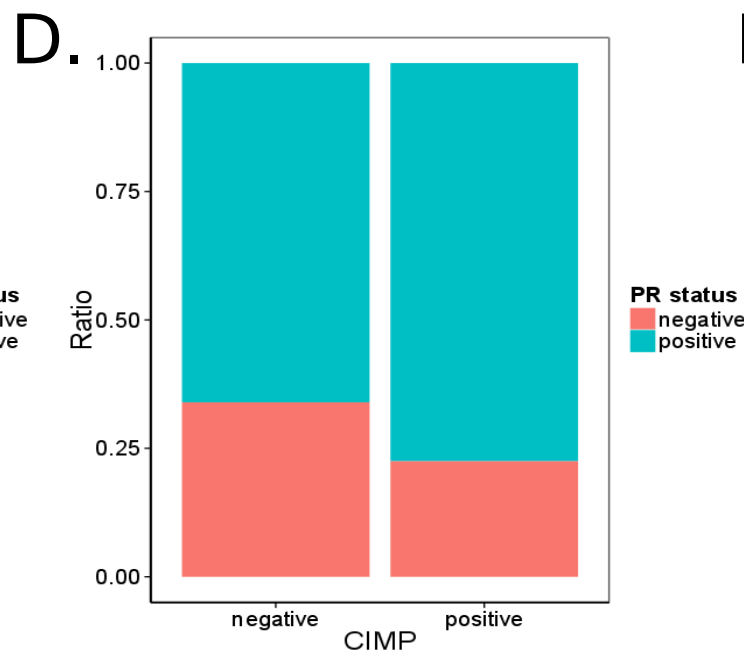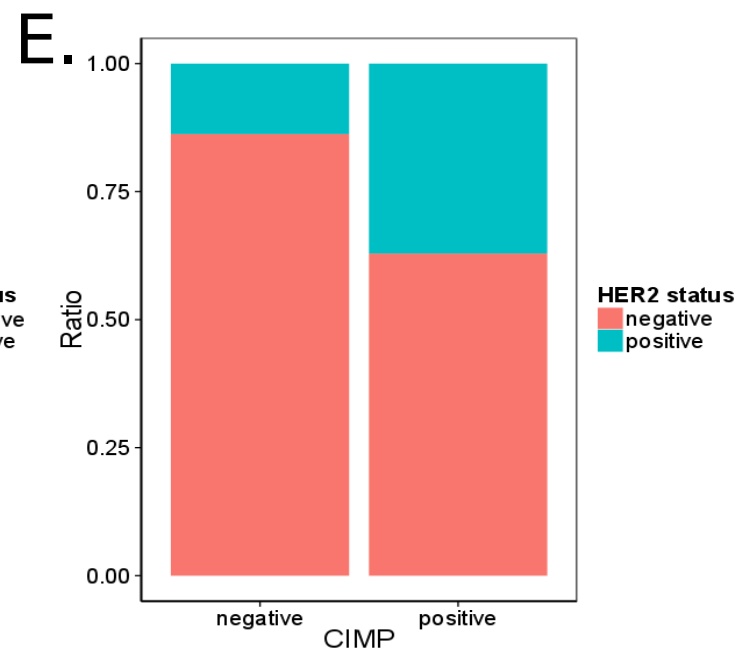

Supplement: Additional file 7 — Association between CIMP and clinical annotations. Panel A. Association between CIMP and age: distribution of patients’ age given their CIMP phenotype in each tissue. Panel B. Association between CIMP and MSI in colon: ratio of MSI-positive and MSI-negative patient given the CIMP phenotype in the colon. Panel C. Association between CIMP and ER status in breast: ratio of ER-positive patients given the CIMP phenotype in the breast. Panel D. Association between CIMP and PR status in the breast: ratio of PR-positive patients given the CIMP phenotype in the breast. Panel E. Association between CIMP and HER2 status in the breast: ratio of HER2-positive patients given the CIMP phenotype in the breast. (PDF 86 kb) [file 40246_2015_48_MOESM7_ESM.pdf]
