# Supplementary material for: Integrative DNA methylation and gene expression analysis to assess the universality of the CpG island methylator phenotype
Source: Hum Genomics. 2015 Oct 13;9:26. doi: 10.1186/s40246-015-0048-9 (PMC4603341; doi:10.1186/s40246-015-0048-9)
Supplement: Additional file 2 — Stability of CIMP clusters with respect to the size of the CIMP signature. Robustness of cluster assignment for each sample (columns) as a function of the proportion of variant CGIs kept to define the CIMP signature, from 1 to 10 % (rows) and given the number of CIMP clusters considered (left panels: K = 2, right panels: K = 3, yellow = CIMP-positive, blue = CIMP-negative, black = CIMP-low) for bladder (panel A/B), breast (panel C/D), colon (panel E/F), lung (panel G/H), stomach (panel I/J). Panel K. Table summarizing the stability of the cluster assignments for each tissue and different number of CIMP clusters considered. (PDF 381 kb) [file 40246_2015_48_MOESM2_ESM.pdf]

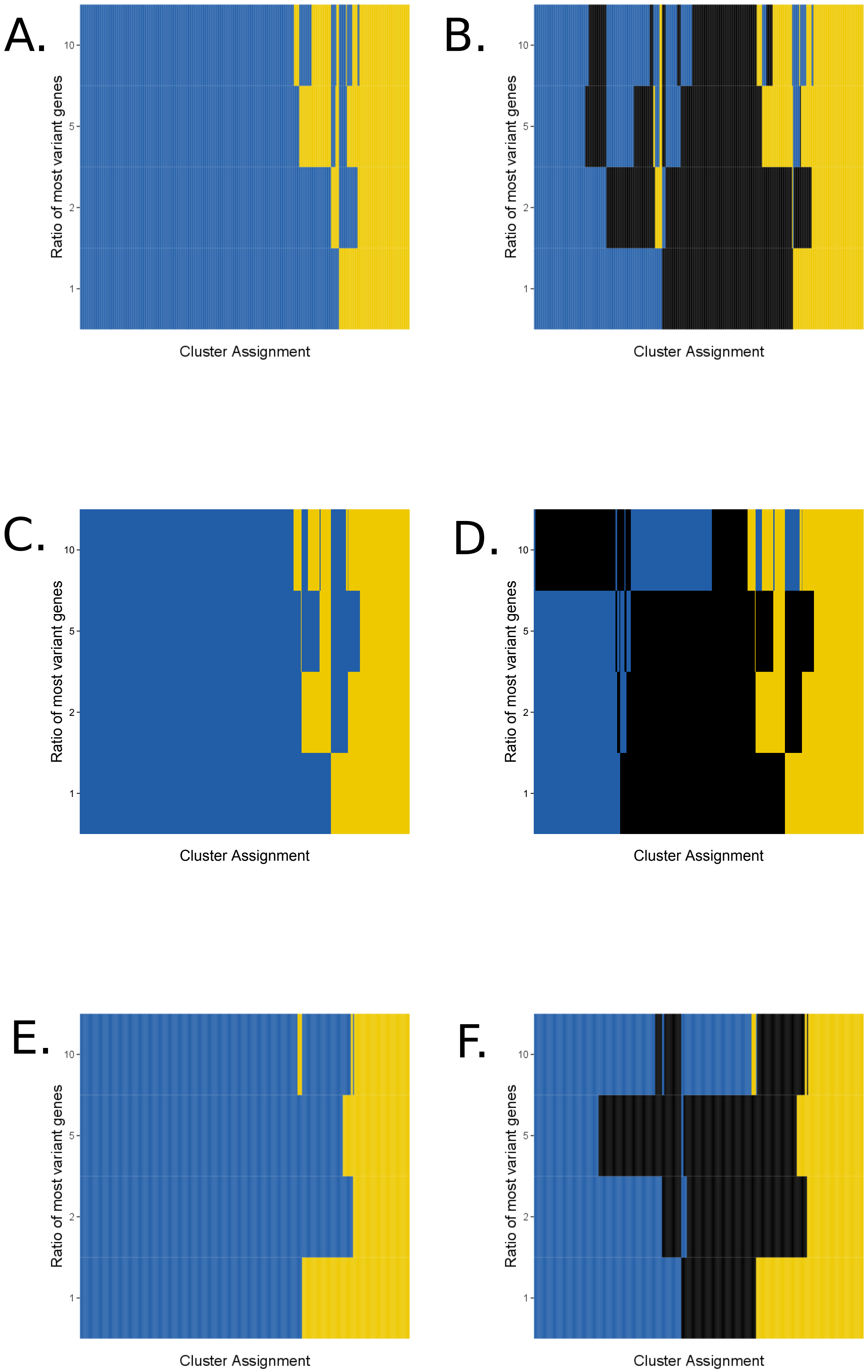
A.
B.
C.
D.
E.
F.
Ratio of most variant genes
10
5
2
1
Cluster Assignment

G.

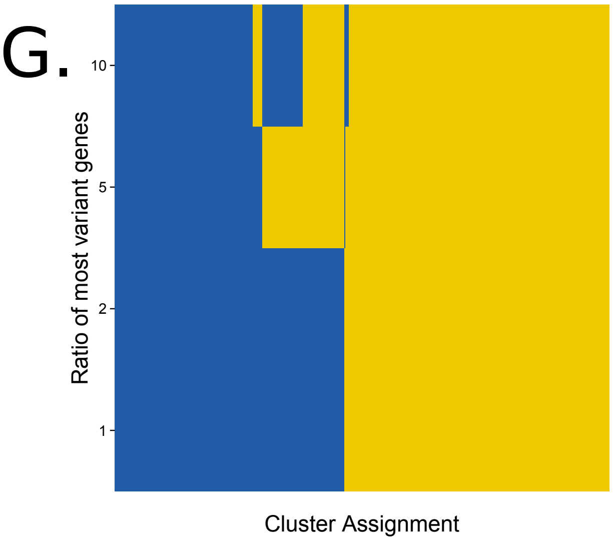

H.

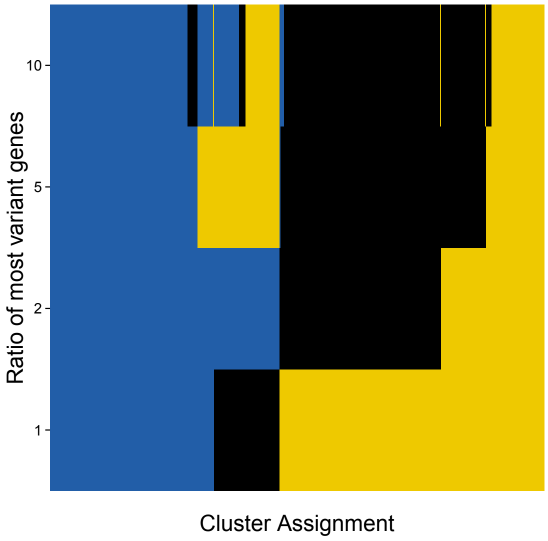

I.

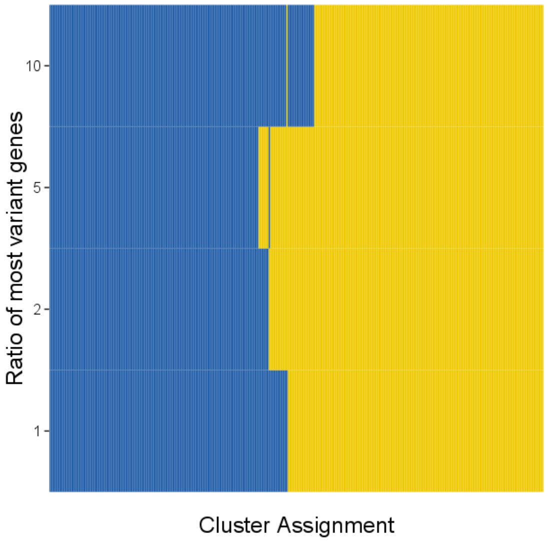

J.

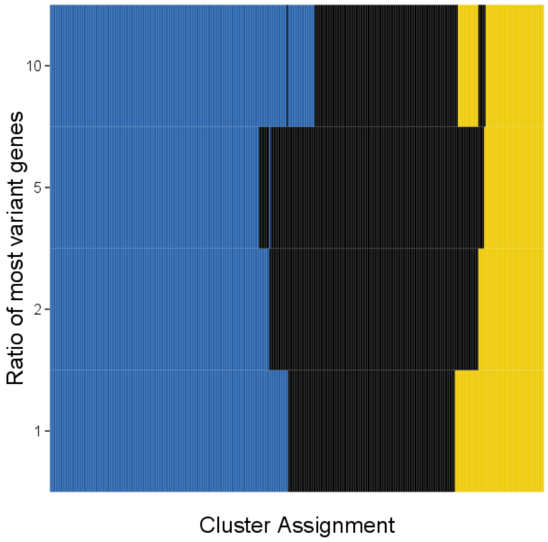

K.

Tissue	K=2	K=3
**Bladder**	80%	50%
**Breast**	80%	26%
**Colon**	82%	36%
**Lung**	81%	38%
**Stomach**	89%	82%
