# Supplementary material for: Integrative DNA methylation and gene expression analysis to assess the universality of the CpG island methylator phenotype
Source: Hum Genomics. 2015 Oct 13;9:26. doi: 10.1186/s40246-015-0048-9 (PMC4603341; doi:10.1186/s40246-015-0048-9)
Supplement: Additional file 3 — Supplementary methods. (PDF 153 kb) [file 40246_2015_48_MOESM3_ESM.pdf]

Supplementary methods for: Integrative DNA methylation and gene expression profiles to assess the universality of the CpG island methylator phenotype

Matahi Moarii, Fabien Reyal and Jean-Philippe Vert

1 Predicting CIMP status from gene expression profiles

To predict CIMP using gene expression profiles, we perform logistic regression using a lasso penalty [1] with different settings described below. We consider K different tissues ($K = 5$ in the manuscript: bladder, breast, colon, lung and stomach), and for each tissue $k = 1, \ldots, K$ we have n_k samples described for each sample $i = 1, \ldots, n_k$ by a p-dimensional vector x_i^k (the expression levels of p genes) and a response $y_i^k \in \{-1, +1\}$ (the CIMP class of the sample).

In all cases, we learn a linear logistic regression model for each tissue parametrized by a vector $\beta_k \in \mathbb{R}^p$ that models the probability of the CIMP class Y of a sample $X \in \mathbb{R}^p$ of the k-th tissues as:

$$P(Y \mid X, \beta_k) = \frac{1}{1 + \exp\left(-Y \beta_k^\top X\right)} . \quad (1)$$

The likelihood of a model parameter β_k on the observed samples of the k-th class is therefore:

$$\ell_k(\beta_k) = \sum_{i=1}^{n_k} -\log\left(1 + e^{-y_i^k \beta_k^\top x_i^k}\right) .$$

1.1 Tissue-specific lasso

Our first strategy is to consider the different tissues independently from each other, and fit a lasso-regularized logistic regression on each tissue. This amounts to solving, for $k = 1, \ldots, K$:

$$\hat{\beta}_k = \arg\min_{\beta_k \in \mathbb{R}^p} \left\{-\ell_k(\beta_k) + \lambda \|\beta_k\|_1\right\} ,$$

where ℓ_k is the log-likelihood for the k-th tissue (1) and $\|\beta_k\|$ is the ℓ_1 norm of β_k, namely, the sum of the absolute values of its coefficients. The resulting models will be sparse, i.e., will automatically select a few genes whose expressions are combined to predict the CIMP status. Note that, since models are learned independently on each tissue, the genes selected in each tissue are likely to be different.

1.2 Combined Lasso

Our second strategy is to fit a unique model for all tissues, by pooling all samples together. This amounts to estimating a single $\hat{\beta} = \hat{\beta}_1 = \ldots = \hat{\beta}_K$ my solving:

$$\hat{\beta} = \arg \min_{\beta \in \mathbb{R}^p} \left\{ -\sum_{k=1}^{K} \ell_k(\beta) + \lambda \|\beta\|_1 \right\} .$$

In this strategy, the models for the different tissues not only share the same genes, but also the same weights: they are identical.

1.3 Group Lasso

Our third strategy is to fit K different models jointly, sharing information across the tissues without imposing the models to be identical. For that purpose, we follow the group lasso strategy [2] and solve the problem:

$$\left(\hat{\beta}_1, \ldots, \hat{\beta}_K\right) = \arg \min_{(\beta_1, \ldots, \beta_K) \in \mathbb{R}^{pK}} \left\{ -\sum_{k=1}^{K} \ell_k(\beta_k) + \lambda \sum_{j=1}^{p} \left(\sum_{l=1}^{K} \beta_{lj}^2 \right)^{\frac{1}{2}} \right\} ,$$

The group lasso penalty constrains the models for the different tissues to be sparse and select the same genes; however the weights of the genes can differ between tissues.

1.4 Random strategy

As a baseline we assess the performance of a simple strategy that predicts the CIMP class independently from the measured gene expression. Given the imbalanced proportion of CIMP in each datasets, we define the “random” predictor as a predictor that always predicts the majority class.

1.5 Evaluation

The statistical significance of how better a gene expression based predictor is compared to the “random” predictor was calculated using a Student t-test, based on the accuracies measured in 3-fold cross-validation repeated 100 times.

1.6 Gene signature

To determine the genetic predictive signature, genes were ranked according to their frequency of occurrence in the optimal lasso estimator signature, averaged over the different folds and repeats [3]. Genes whose frequency was superior to 50% were selected.

References

[1] Tibshirani, R.: Regression shrinkage and selection via the lasso. J. R. Stat. Soc. Ser. B **58**(1), 267–288 (1996)

[2] Meier, L., van de Geer, S., Bühlmann, P.: The group lasso for logistic regression. J. R. Stat. Soc. Ser. B **70**(1), 53–71 (2008)

[3] Meinshausen, N., Bühlmann, P.: Stability selection. J. R. Stat. Soc. Ser. B **72**(4), 417–473 (2010)
